# A cross-sectional analysis of the relationship between the non-high density to high density lipoprotein cholesterol ratio (NHHR) and kidney stone risk in American adults

**DOI:** 10.1186/s12944-024-02150-9

**Published:** 2024-05-27

**Authors:** Yuan-Zhuo Du, Qian-Xi Dong, Hong-Ji Hu, Biao Guo, Yi-He Li, Ji Zhang, Fu-Chun Li, Ju Guo

**Affiliations:** 1 Department of Urology, The First Affiliated Hospital, Jiangxi Medical College, Nanchang University, Nanchang, 330000 Jiangxi Province China; 2 Jiangxi Institute of Urology, Nanchang, 330000 Jiangxi Province China

**Keywords:** NHHR, Kidney stones, NHANES, Cross-sectional study

## Abstract

**Background:**

Recent interest in the Non-High Density to High Density Lipoprotein Cholesterol ratio (NHHR) has emerged due to its potential role in metabolic disorders. However, the connection between NHHR and the development of kidney stones still lacks clarity. The primary goal of this research is to explore how NHHR correlates with kidney stone incidence.

**Methods:**

An analysis was conducted on the data collected by the National Health and Nutrition Examination Survey (NHANES) between 2007 and 2018, focusing on adults over 20 years diagnosed with kidney stones and those with available NHHR values. Employing weighted logistic regression and Restricted Cubic Spline (RCS) models, NHHR levels’ correlation with kidney stone risk was examined. Extensive subgroup analyses were conducted for enhanced reliability of the findings.

**Results:**

The findings indicate a heightened kidney stone risk for those at the highest NHHR levels relative to those at the lowest (reference group). A notable non-linear correlation of NHHR with kidney stone incidence has been observed, with a significant *P*-value (< 0.001), consistent across various subgroups.

**Conclusion:**

A clear link exists between high NHHR levels and increased kidney stone risk in the American adult population. This study highlights NHHR’s significance as a potential indicator in kidney stone formation.

## Background

 The escalating global incidence of kidney stones, which has been documented extensively in recent literature [1–3], poses significant challenges to patient well-being and imposes considerable economic strains on healthcare systems worldwide [4–6]. This upward trend, coupled with the stones’ recurrent nature, underscores the critical need for enhanced preventive strategies [7, 8]. The concurrent surge in metabolic disorders, including obesity and diabetes, has paralleled an increase in kidney stone prevalence, signaling a worldwide exacerbation of metabolic health issues [2, 9, 10].

The development of kidney stones is understood to be a multifaceted combination of genetic, environmental, dietary, lifestyle, and metabolic influences. These elements can alter the urine’s chemical composition and pH, indirectly influencing stone risk [11–18]. In particular, dysregulated lipid metabolism may precipitate stone formation through modifications in urinary calcium and oxalate levels, and pH, thereby facilitating crystal nucleation and growth [17–19].

Despite significant advancements in understanding the metabolic determinants of kidney stone formation, the role of specific lipid parameters, particularly the Non-High Density to High Density Lipoprotein Cholesterol ratio (NHHR) is recognized for its enhanced precision in predicting metabolic syndromes and insulin resistance [20], with its elevated levels correlating with increased risks of various conditions, including abdominal aortic aneurysm, depression, and periodontitis [21–23]. However, the association between NHHR and the incidence of kidney stones has not been thoroughly investigated. Therefore, it is hypothesized that elevated NHHR levels directly correlate to a greater likelihood of developing kidney stones.

This investigation seeks to delineate the potential link between NHHR and the incidence of kidney stones, analyzing epidemiological data to shed light on this association. By examining the unique role of NHHR in kidney stone pathogenesis, the study endeavors to provide novel insights into the mechanisms underlying stone formation. Furthermore, the elucidation of NHHR’s involvement in kidney stones may hold implications for the development of targeted preventive and therapeutic strategies, thereby contributing to the advancement of patient care and management in this prevalent and burdensome urological condition.

## Methods

### Study population

The study utilizes information collected from the National Health and Nutrition Examination Survey (NHANES), which samples different groups across the U.S. in stages to evaluate their health and nutrition regularly. NHANES meticulously collects a wide array of data relevant to health and nutrition through annual surveys of approximately five thousand Americans, capturing detailed information on demographics, socioeconomic status, dietary patterns, and health conditions. Data collection is conducted through face-to-face interviews and comprehensive physical examinations, which include physiological measurements and laboratory tests. All participating individuals provided informed consent, and the survey’s methodology, including the informed consent process, received approval from the Ethics Committee at the National Center for Health Statistics.

**Fig. 1:**
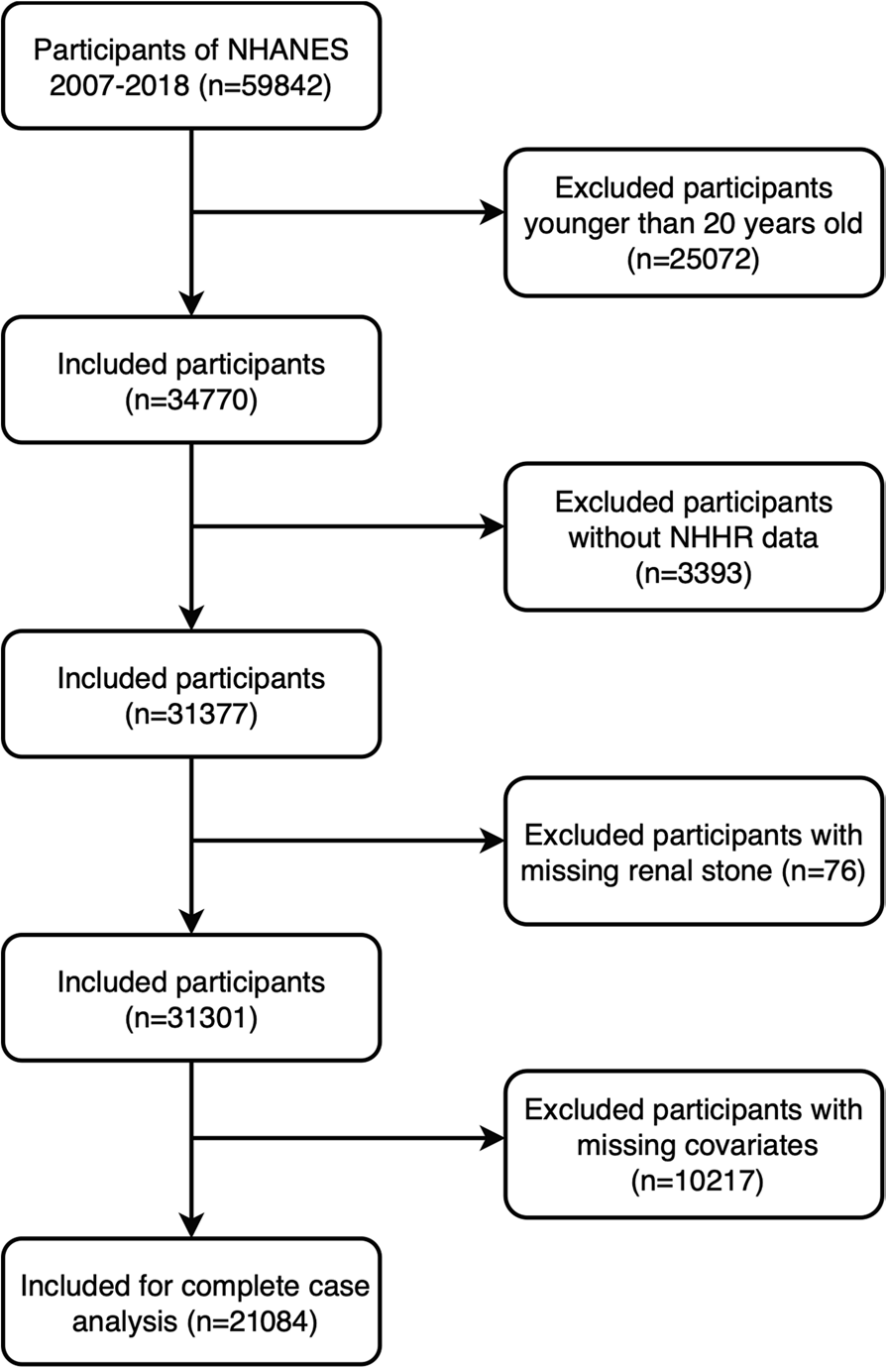
The participant flow diagram

Data spanning from 2007 to 2018 for a cross-sectional analysis were extracted from the NHANES database. The selection process for eligible research subjects, from an initial pool of 59,842 candidates, adhered to specific inclusion and exclusion criteria. Exclusion criteria eliminated individuals younger than twenty years (*n* = 25,072), those without the necessary information for NHHR calculation (*n* = 3,393), those without a documented history of kidney stones (*n* = 76), and participants lacking key covariate information (*n* = 10,217). Ultimately, 21,084 participants satisfied the criteria and were incorporated into the final analysis, as detailed in Fig. 1.

### Assessment of NHHR

This research calculates the NHHR from the subjects’ lipid profiles. The NHHR is computed as Total Cholesterol (TC) minus High-Density Lipoprotein Cholesterol (HDL-C), all divided by HDL-C. Non- High-Density Lipoprotein Cholesterol (Non-HDL-C) equals TC minus HDL-C.

### Assessment of kidney stones

This study assessed the participants’ prior instances with kidney stones by querying, “Did you previously experience kidney stones?” Data on kidney stones history derived from participants’ self-reports, validated by prior research confirming the accuracy of self-reported kidney stone histories [24]. Respondents affirming a history of kidney stones with a ‘yes’ were thus classified as having a history of the condition. Additionally, the data collected represents the prevalence of kidney stones among the participants, indicating whether they have ever had kidney stones at any point in their life, rather than the incidence of new cases during the study period.

### Covariates

In this investigation, a comprehensive array of covariates associated with NHHR status and kidney stone risk was incorporated. These variables span three major categories: demographics, lifestyle habits, and health indicators. Population characteristics encompassed age, sex, ethnicity, marital status, education level, along with poverty ratio. Lifestyle factors considered were alcohol intake, smoking status, sedentary behavior, and physical activity levels. Alcohol consumption was categorized as never (fewer than 12 lifetime drinks), former (12 or more drinks annually, excluding the past year), and current (12 or more lifetime drinks, including at least one in the preceding year) [25]. Smoking was determined by a history of consuming over 100 cigarettes. Sedentary duration was defined as daily sitting time excluding sleep, categorized as under 5 h–5 h and more. Physical activity was assessed based on periods of moderate to vigorous activity lasting at least 10 consecutive minutes, aside from routine work and commuting tasks, with inactivity defined as less than 10 min of such activity [26]. Health indicators included Body Mass Index (BMI), estimated Glomerular Filtration Rate (eGFR), and total calcium levels. The data also covered medication usage such as thiazide diuretics, loop diuretics, and antihyperlipidemic agents, as well as history of gout, diabetes, hypertension, and cardiovascular diseases (CVD), collected via standardized questionnaires and clinical evaluations.

### Statistical analysis

In this study, NHANES’ standard sample weighting guidelines were adhered to ensure the representativeness of the sample, applying necessary adjustments to weights. Specifically, weights for the 12-year, two-day dietary samples were adjusted to equal the sample weights for those two days, divided by six. Statistical analyses included both continuous (means and standard errors) and categorical (counts and ratios) variables, using weighted logistic regression and chi-square tests, respectively.

Investigations into NHHR’s impact on kidney stone formation utilized multiple logistic regression analysis. Odds ratios (ORs) and 95% confidence intervals (CIs) for NHHR got calculated, considering it as both a continuous and categorical variable. NHHR got divided into quartiles for analysis, using the bottom quartile for the baseline. Crude model was unadjusted, whereas Model 1 included adjustments for a variety of factors, such as age, sex, ethnicity, marital status, education level, along with poverty ratio. Model 2 additionally took into account factors including smoking, alcohol consumption behavior, BMI, total cholesterol, dietary cholesterol intake, eGFR, sedentary time, physical activity level, serum total calcium levels, thiazide diuretics, loop diuretics, and antihyperlipidemic agents, as well as history of gout, hypertension, diabetes, and CVD. Additionally, a restricted cubic spline (RCS) regression was utilized for examining dose-response interactions involving NHHR with kidney stone risk, with subgroup analyses assessing the robustness of the findings across various population factors. Statistical evaluations were conducted in R (version 4.3.2), maintaining a significance level of *P* < 0.05.

## Results

### Characteristics of participants

In the investigation, information from 21,084 participants covering six NHANES cycles from 2007 to 2018 was analyzed, categorizing them into two groups based on their history of kidney stones. Table 1 outlines demographic details for participants, with an average age of 47.24 ± 0.28 years. Among these individuals, 2,056 reported a history of kidney stones. Notably, those with kidney stones had a significantly higher NHHR than those without (3.13 ± 0.05 vs. 2.93 ± 0.02). The kidney stone cohort was characterized by a more advanced average age, a greater proportion of males and non-Hispanic whites, and a greater likelihood of being single (including divorced, separated, or widowed). Additionally, they exhibited higher BMI values and a higher prevalence of smoking or former drinking habits. This group also demonstrated a tendency towards a sedentary lifestyle, was more likely to use thiazide diuretics, loop diuretics, and antihyperlipidemic agents, and had higher rates of gout, hypertension, diabetes, and CVD. Further distinctions included lower eGFR, decreased levels of HDL-C, and lower TC values.

**Table 1:** Baseline characteristics of the study population

Characteristic	Overall (*n* = 21,084)	Non-stone formers (*n* = 19,028)	Stone formers (*n* = 2056)	*P*-value
Age, mean (SE)	47.24(0.28)	46.59(0.30)	53.27(0.48)	< 0.0001
Age strata, n (%)				< 0.0001
20–39	6957(36.87)	6567(38.52)	390(21.50)	
40–59	7052(37.59)	6347(37.22)	705(41.06)	
≥60	7075(25.54)	6114(24.27)	961(37.44)	
Sex, n (%)				< 0.001
Female	10,901(51.61)	10,007(52.44)	894(43.89)	
Male	10,183(48.39)	9021(47.56)	1162(56.11)	
Race, n (%)				< 0.0001
Mexican American	2994(8.31)	2745(8.59)	249(5.68)	
Non-Hispanic White	9562(68.75)	8372(67.72)	1190(78.37)	
Non-Hispanic Black	4251(10.16)	3987(10.63)	264(5.76)	
Other Hispanic	2067(5.34)	1856(5.38)	211(5.00)	
Other Race	2210(7.44)	2068(7.68)	142(5.19)	
Marital status, n (%)				< 0.0001
Divorced/Separated/Widowed	4588(17.70)	4056(17.44)	532(20.14)	
Married/Living with a partner	12,762(63.63)	11,424(62.89)	1338(70.54)	
Never married	3734(18.67)	3548(19.67)	186(9.33)	
Education levels, n (%)				0.99
High school and below	9310(36.36)	8372(36.36)	938(36.34)	
Above high school	11,774(63.64)	10,656(63.64)	1118(63.66)	
Poverty ratio, n (%)				0.29
<1.3	6406(20.91)	5782(20.98)	624(20.25)	
1.3–3.5	7958(34.66)	7161(34.43)	797(36.85)	
>3.5	6720(44.43)	6085(44.60)	635(42.91)	
BMI, n (%)				< 0.0001
<18.5	290(1.31)	279(1.36)	11(0.83)	
18.5-24.99	5534(27.75)	5148(28.55)	386(20.31)	
25-29.99	6931(32.71)	6238(32.71)	693(32.76)	
≥30	8329(38.22)	7363(37.38)	966(46.09)	
Smoke, n (%)				0.003
No	11,739(56.60)	10,747(57.10)	992(51.87)	
Yes	9345(43.40)	8281(42.90)	1064(48.13)	
Alcohol user, n (%)				0.003
Never	2851(10.59)	2578(10.51)	273(11.32)	
Former	3360(12.82)	2919(12.41)	441(16.64)	
Now	14,873(76.60)	13,531(77.08)	1342(72.04)	
Recreational activity, n (%)				< 0.0001
Inactive	10,645(43.69)	9459(42.90)	1186(51.04)	
Active	10,439(56.31)	9569(57.10)	870(48.96)	
Sitting time, n (%)				0.06
<5	8438(36.72)	7644(37.02)	794(33.83)	
≥5	12,646(63.28)	11,384(62.98)	1262(66.17)	
Hypertension, n (%)				< 0.0001
No	13,403(68.48)	12,401(70.13)	1002(53.10)	
Yes	7681(31.52)	6627(29.87)	1054(46.90)	
Diabetes, n (%)				< 0.0001
No	17,887(88.74)	16,352(89.94)	1535(77.50)	
Borderline	491(1.81)	426(1.61)	65(3.66)	
Yes	2706(9.46)	2250(8.45)	456(18.84)	
CVD, n (%)				< 0.001
No	20,416(97.78)	18,478(97.94)	1938(96.20)	
Yes	668(2.22)	550(2.06)	118(3.80)	
Gout, n (%)				< 0.0001
No	20,091(95.97)	18,221(96.38)	1870(92.07)	
Yes	993(4.03)	807(3.62)	186(7.93)	
Loop diuretic user, n (%)				< 0.0001
No	20,369(97.58)	18,439(97.86)	1930(94.98)	
Yes	715(2.42)	589(2.14)	126(5.02)	
Thiazide user, n (%)				< 0.001
No	19,942(95.54)	18,030(95.78)	1912(93.24)	
Yes	1142(4.46)	998(4.22)	144(6.76)	
Antihyperlipidemic agent user, n (%)				< 0.0001
No	16,579(81.37)	15,184(82.36)	1395(72.16)	
Yes	4505(18.63)	3844(17.64)	661(27.84)	
Serum total calcium (mmol/L), mean (SE)	2.35(0.00)	2.35(0.00)	2.35(0.00)	0.3
eGFR (mL/min), mean (SE)	94.40(0.35)	95.20(0.37)	86.98(0.61)	< 0.0001
Dietary cholesterol (mg), mean (SE)	294.06(2.58)	292.55(2.66)	308.20(8.28)	0.07
Total Cholesterol (mg/dL), mean (SE)	194.41(0.58)	194.67(0.60)	191.95(1.29)	0.04
HDL-C (mg/dL), mean (SE)	53.36(0.23)	53.72(0.24)	49.99(0.52)	< 0.0001
NHHR, mean (SE)	2.95(0.02)	2.93(0.02)	3.13(0.05)	< 0.001

*NHHR* Non-HDL-C and HDL-C ratio, *HDL-C* High-density lipoprotein cholesterol, *eGFR* Estimated Glomerular Filtration Rate, *BMI* Body mass index, *CVD* Cardiovascular disease

### Association between NHHR and kidney stones

**Fig. 2:**
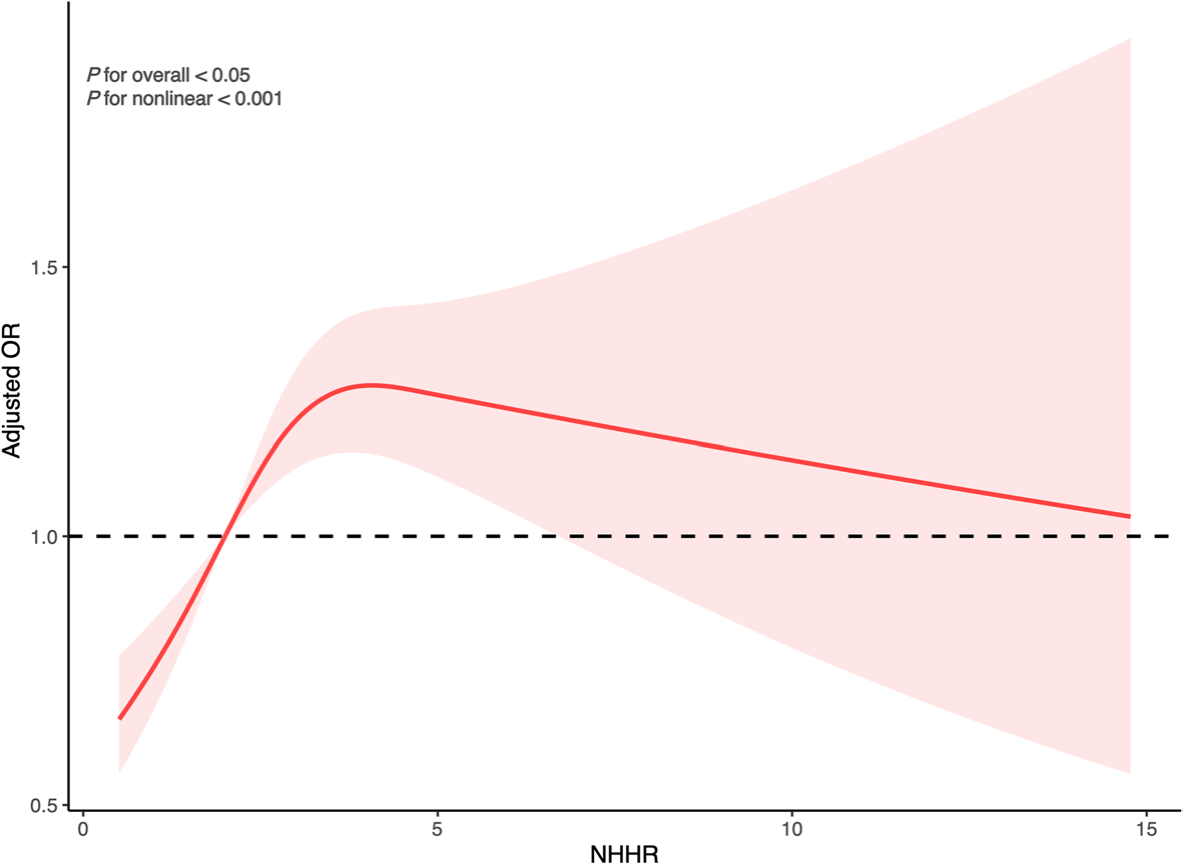
Illustrates the correlation between NHHR and the incidence of kidney stones. The ORs, depicted by solid lines, were adjusted for age, sex, ethnicity, marital status, education levels, poverty ratio, BMI, smoking, alcohol user, recreational activity, sitting time, eGFR, serum total calcium levels, thiazide diuretics, loop diuretics, antihyperlipidemic agents, gout, hypertension, diabetes, CVD, TC, and Dietary cholesterol. Corresponding 95% CIs are represented by shaded areas

Table 2 illustrates a significant positive correlation between NHHR as a continuous variable and the risk of kidney stones. Initial analysis without adjustment indicated that a one-unit increase in NHHR correlates with a 9% rise in kidney stone risk (95% CI: 1.05–1.13, *P* < 0.0001). This relationship persisted in Model 2, which accounted for all examined covariates, with an OR of 1.05 (95% CI: 1.00-1.11, *P* = 0.04). Furthermore, in the quartile-based model with full adjustments, the risk progressively escalated across the NHHR quartiles compared to the lowest quartile: the second quartile saw a 30% increase in risk (95% CI: 1.04–1.63, *P* = 0.02), the third quartile 43% (95% CI: 1.10–1.85, *P* = 0.01), and the fourth quartile 39% (95% CI: 1.07–1.80, *P* = 0.01). The analysis employing a RCS regression model suggested a potential dose-response relationship, manifested as a curve with a peak at an NHHR inflection point of 4.09 (Fig. 2).

**Table 2:** Association of the NHHR with kidney stone

Exposure	Crude model			Model 1		Model 2	
	OR (95% CI)		*P* value	OR (95% CI)	*P* value	OR (95% CI)	*P* value
NHHR	1.09(1.05,1.13)		< 0.0001	1.06(1.02,1.10)	0.01	1.05(1.00,1.11)	0.04
NHHR quartile							
Quartile1	1 (Ref.)			1 (Ref.)		1 (Ref.)	
Quartile2	1.40(1.14,1.71)		0.001	1.33(1.09,1.64)	0.01	1.30(1.04,1.63)	0.02
Quartile3	1.61(1.29,2.02)		< 0.0001	1.46(1.16,1.83)	0.001	1.43(1.10,1.85)	0.01
Quartile4	1.59(1.30,1.95)		< 0.0001	1.43(1.17,1.75)	< 0.001	1.39(1.07,1.80)	0.01
* P* for trend			< 0.0001		< 0.001		0.01

Crude model: unadjusted model

Model 1: Adjusted for age, sex, race, education levels, marital status, poverty ratio

Model 2: Additionally adjusted for BMI, smoking, alcohol user, recreational activity, sitting time, eGFR, serum total calcium levels, thiazide diuretics, loop diuretics, antihyperlipidemic agents, gout, hypertension, diabetes, CVD, TC and Dietary cholesterol

*NHHR* Non-HDL-C and HDL-C ratio, *OR* odds ratio, *CI* confidence interval

### Subgroup analyses

**Fig. 3:**
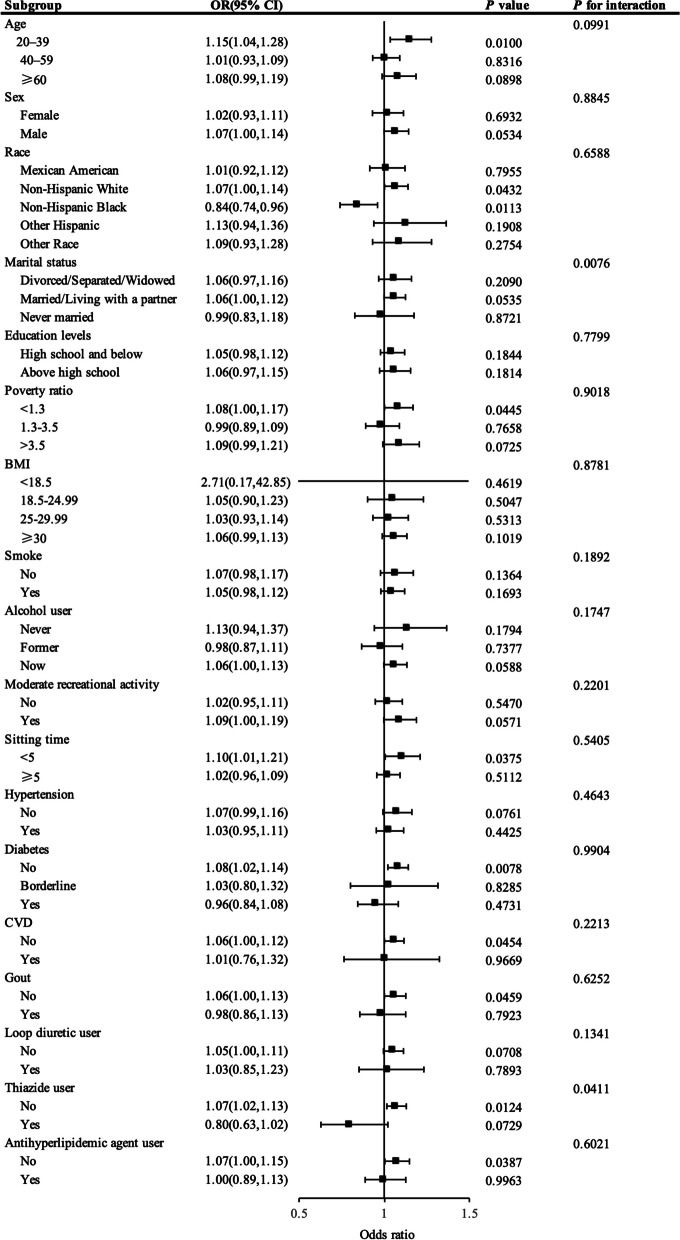
Presents a stratified analysis of kidney stones and NHHR

Figure 3 presents the outcomes of a stratified analysis, demonstrating the differential associations of NHHR with kidney stone risk across various demographic segments. Notably, the strongest association was identified in individuals aged 20 to 40 years, with an OR of 1.15 (95% CI: 1.04–1.28, *P* = 0.01). Notable correlations were detected in non-Hispanic whites (OR = 1.07, 95% CI: 1.00-1.14) and non-Hispanic blacks (OR = 0.84, 95% CI: 0.74–0.96). Lower income levels showed a meaningful association (OR = 1.08, 95% CI: 1.00-1.17), and non-sedentary lifestyles also demonstrated a significant correlation (OR = 1.10, 95% CI: 1.01–1.21). Individuals without prior occurrences of gout (OR = 1.06, 95% CI: 1.00-1.11), CVD (OR = 1.06, 95% CI: 1.00-1.12) or diabetes (OR = 1.08, 95% CI: 1.02–1.14) also exhibited significant associations. Participants with no use of thiazide diuretics (OR = 1.07, 95% CI: 1.02–1.13) or antihyperlipidemic agents (OR = 1.07, 95% CI: 1.00-1.15) showed associations with NHHR levels, with all associations yielding *P*-values below 0.05. However, this relationship appears to be inversely correlated among non-Hispanic blacks. Additionally, a significant interaction was identified between marital status, the use of thiazide diuretics, and NHHR levels, affecting kidney stone risk and demonstrating the complexity these relationships, with a *P*-value < 0.05.

## Discussion

This investigation establishes a notable positive association between the NHHR and the incidence of kidney stones. This correlation persists across various model adjustments and analytical methodologies. Analysis through RCS regression models indicates a potential nonlinear relationship, with inflection points suggesting a more pronounced risk increase at certain NHHR levels. These findings bolster the hypothesis that NHHR, indicative of lipid metabolic activity, may influence kidney stone formation.

In this study, the stone group was observed to have a higher number of male participants compared to the non-stone group. This difference in gender ratio may impact the comparison of eGFR across groups. Existing research indicates that at equivalent levels of creatinine, eGFR is generally higher in males than in females. This difference could be attributed to greater muscle mass in males, leading to higher creatinine production. Therefore, gender is a significant factor to adjust for when using creatinine-based eGFR formulas. In the stone group, the higher proportion of males may lead to lower average calculated eGFR values, particularly when gender has not been appropriately adjusted for. Future research should further explore how gender affects the assessment of renal function in stone patients and consider incorporating more gender adjustment factors into the eGFR formula to enhance assessment accuracy.

Historically viewed as an isolated condition, recent research links kidney stones to systemic metabolic disturbances, notably lipid dysregulation [27–29]. Several studies have connected lipid anomalies with urolithiasis risk. Elevated triglycerides and cholesterol, for instance, may modify the urinary composition in individuals without stones, potentially facilitating stone genesis [30]. Similarly, links between obesity, metabolic changes, and stone recurrence have been identified [31], with heightened serum triglyceride levels correlating with increased urolithiasis risk [28]. Lipoproteins in their oxidized form might catalyze stone formation by promoting oxidative stress and inflammatory responses, altering the lithogenic environment [32].

NHHR mirrors the balance between HDL-C and non-HDL-C, lipoproteins with divergent roles [33]. An escalated NHHR may signify lipid metabolic imbalances. To further elaborate on the underlying mechanisms, it is imperative to consider the biochemical properties of HDL and non-HDL cholesterol. HDL-C is lauded for its cardio-protective effects [34] and is pivotal in the process of reverse cholesterol transport [35], which involves the removal of surplus cholesterol from peripheral tissues and atheromas and transports it back to the liver for excretion. whereas non-HDL-C elevation is linked to atherogenesis [36], it is known to contribute to the buildup of plaques in arteries [37], potentially exacerbating conditions conducive to kidney stone formation by affecting renal blood flow and calcification processes.

Therefore, NHHR rise could indicate a pro-inflammatory, pro-oxidative state associated with stone pathogenesis, as observed in metabolic syndrome [9]. Additionally, the influence of dietary factors on NHHR levels and kidney stone risk cannot be underestimated. Diets high in saturated fats can increase levels of harmful cholesterol [38–40], potentially worsening NHHR imbalances. On the other hand, diets rich in fruits, vegetables, and fibers can improve HDL-C levels and overall lipid profiles [41, 42], thereby mitigating the risk associated with high NHHR. These dietary effects are crucial to consider when developing preventive strategies against kidney stones, especially in populations predisposed to high cholesterol levels.

Considering NHHR’s influence on kidney stone risk necessitates acknowledging cholesterol’s multifaceted role within biological systems. Beyond cell membrane constitution, cholesterol is pivotal in cell signaling and inflammation [43, 44]. Hence, NHHR elevations might reflect cellular and metabolic shifts predisposing to crystallization. Furthermore, lipid dysregulation may alter urine’s chemical composition and pH, influencing stone formation [32]. Future research should delve into the biochemical and molecular dynamics of lipid metabolism and urolithiasis, aiding in novel preventative and therapeutic strategy development, possibly through dietary and lifestyle modifications targeting lipid management.

Moreover, the outcomes of this study are profoundly relevant to clinical practice, especially concerning patient care and the formulation of disease prevention strategies. By uncovering the link between NHHR and the risk of kidney stones, this research offers clinicians a potential biomarker for identifying high-risk patients in routine practice and implementing targeted preventive actions. Doctors, for example, might adjust treatment protocols and lifestyle advice based on a patient’s NHHR levels. For those with elevated NHHR, recommendations could include reducing intake of saturated fats and cholesterol, boosting high-density lipoprotein levels, and enhancing lipid metabolism through diet and exercise. Moreover, increasing monitoring frequency for these patients—regularly checking their lipid profiles and kidney function—could lead to early problem detection and intervention.

Additionally, the study underscores the necessity of a holistic treatment approach, recommending that the link between lipid metabolism and kidney stone risk be considered in patient management. This consideration is especially vital in preventing kidney stone recurrence, as managing lipid levels could reduce stone formation and improve overall health. Ultimately, these insights encourage clinicians to take a more comprehensive and personalized approach when treating kidney stone patients. This not only improves immediate clinical outcomes but also contributes to long-term health maintenance, reducing kidney stone formation and recurrence and significantly enhancing the patient’s quality of life.

### Study strengths and limitations

This research employed the comprehensive and extensive NHANES database, which boasts a nationally representative sample and meticulous data collection methods. These attributes laid a solid foundation for t investigating how NHHR relates to the risk of kidney stones. Utilizing multivariate logistic regression and RCS regression models, the study revealed a potential dose-response correlation between NHHR and the likelihood of kidney stone development, thereby deepening comprehension of this linkage. Moreover, by adjusting for an extensive array of covariates related to demographics, lifestyle habits, and health indicators, the study’s robustness and interpretability were enhanced. Nevertheless, the cross-sectional design constrains capacity to infer causality from these findings. The reliance on self-reported data could introduce recall bias, particularly concerning the history of kidney stones and lifestyle practices. While subgroup analyses offered preliminary insights into varying associations across different populations, they did not encompass all potential interactive factors, indicating that further detailed exploration is necessary in future studies. Given these constraints, forthcoming longitudinal research and more precise data collection methodologies are essential to advance understanding of the NHHR’s relationship with kidney stone risk and to facilitate the formulation of more effective preventative and therapeutic strategies.

## Conclusion

The findings clearly demonstrate a link between elevated NHHR and increased risk of kidney stone formation. The results confirm that elevated NHHR levels significantly correlate with higher kidney stone incidences, supporting NHHR’s potential as an effective biomarker for assessing individual risks. Additionally, these findings deepen understanding of the link between lipid metabolism abnormalities and kidney stone formation. Based on these observations, it is recommended that future prospective studies validate the efficacy and reliability of NHHR as a predictive biomarker, thus providing scientific grounds for the prevention and treatment of kidney stones.

## Data Availability

All data used in this study are available in the NHANES database, accessible at https://www.cdc.gov/nchs/nhanes/index.htm.

## Abbreviations

NHHR: Non-HDL-C and HDL-C ratio
NHANES: National health and nutrition examination survey
RCS: Restricted cubic spline
TC: Total cholesterol
HDL-C: High-density lipoprotein cholesterol
Non-HDL-C: Non-high-density lipoprotein cholesterol
BMI: Body mass index
eGFR: Estimated glomerular filtration rate
CVD: Cardiovascular disease

## References

[1] Scales CD, Smith AC, Hanley JM, Saigal CS (2012). Urologic diseases in America P: prevalence of kidney stones in the United States. Eur Urol.

[2] Kittanamongkolchai W, Vaughan LE, Enders FT, Dhondup T, Mehta RA, Krambeck AE, McCollough CH, Vrtiska TJ, Lieske JC, Rule AD (2018). The changing incidence and presentation of urinary stones over 3 decades. Mayo Clin Proc.

[3] Sorokin I, Mamoulakis C, Miyazawa K, Rodgers A, Talati J, Lotan Y (2017). Epidemiology of stone disease across the world. World J Urol.

[4] Lang J, Narendrula A, El-Zawahry A, Sindhwani P, Ekwenna O (2022). Global trends in incidence and burden of urolithiasis from 1990 to 2019: an analysis of global burden of disease study data. Eur Urol Open Sci.

[5] Raja A, Hekmati Z, Joshi HB (2016). How do urinary calculi influence health-related quality of life and patient treatment preference: a systematic review. J Endourol.

[6] Hyams ES, Matlaga BR (2014). Economic impact of urinary stones. Transl Androl Urol.

[7] Khan SR, Pearle MS, Robertson WG, Gambaro G, Canales BK, Doizi S, Traxer O, Tiselius HG (2016). Kidney stones. Nat Rev Dis Primers.

[8] Peerapen P, Thongboonkerd V (2023). Kidney stone prevention. Adv Nutr.

[9] Wong Y, Cook P, Roderick P, Somani BK (2016). Metabolic syndrome and kidney Stone Disease: a systematic review of literature. J Endourol.

[10] Moftakhar L, Jafari F, Ghoddusi Johari M, Rezaeianzadeh R, Hosseini SV, Rezaianzadeh A (2022). Prevalence and risk factors of kidney stone disease in population aged 40–70 years old in Kharameh cohort study: a cross-sectional population-based study in southern Iran. BMC Urol.

[11] Goldfarb DS, Fischer ME, Keich Y, Goldberg J (2005). A twin study of genetic and dietary influences on nephrolithiasis: a report from the Vietnam era Twin (VET) registry. Kidney Int.

[12] Brikowski TH, Lotan Y, Pearle MS (2008). Climate-related increase in the prevalence of urolithiasis in the United States. Proc Natl Acad Sci U S A.

[13] Ferraro PM, Bargagli M, Trinchieri A, Gambaro G (2020). Risk of kidney stones: influence of dietary factors, dietary patterns, and vegetarian-vegan diets. Nutrients.

[14] Liu M, Wu J, Gao M, Li Y, Xia W, Zhang Y, Chen J, Chen Z, Zhu Z, Chen H (2023). Lifestyle factors, serum parameters, metabolic comorbidities, and the risk of kidney stones: a mendelian randomization study. Front Endocrinol (Lausanne).

[15] Soligo M, Morlacco A, Zattoni F, Valotto C, Beltrami GDEG (2022). Metabolic syndrome and stone disease. Panminerva Med.

[16] Torricelli FC, De SK, Gebreselassie S, Li I, Sarkissian C, Monga M (2014). Dyslipidemia and kidney stone risk. J Urol.

[17] Siener R (2021). Nutrition and kidney Stone Disease. Nutrients.

[18] Khan SR, Glenton PA, Backov R, Talham DR (2002). Presence of lipids in urine, crystals and stones: implications for the formation of kidney stones. Kidney Int.

[19] Howles SA, Thakker RV (2020). Genetics of kidney stone disease. Nat Rev Urol.

[20] Kim SW, Jee JH, Kim HJ, Jin SM, Suh S, Bae JC, Kim SW, Chung JH, Min YK, Lee MS (2013). Non-HDL-cholesterol/HDL-cholesterol is a better predictor of metabolic syndrome and insulin resistance than apolipoprotein B/apolipoprotein A1. Int J Cardiol.

[21] Lin W, Luo S, Li W, Liu J, Zhou T, Yang F, Zhou D, Liu Y, Huang W, Feng Y, Luo J (2023). Association between the non-HDL-cholesterol to HDL- cholesterol ratio and abdominal aortic aneurysm from a Chinese screening program. Lipids Health Dis.

[22] Qi X, Wang S, Huang Q, Chen X, Qiu L, Ouyang K, Chen Y (2024). The association between non-high-density lipoprotein cholesterol to high-density lipoprotein cholesterol ratio (NHHR) and risk of depression among US adults: a cross-sectional NHANES study. J Affect Disord.

[23] Hou K, Song W, He J, Ma Z (2024). The association between non-high-density lipoprotein cholesterol to high-density lipoprotein cholesterol ratio (NHHR) and prevalence of periodontitis among US adults: a cross-sectional NHANES study. Sci Rep.

[24] Curhan GC, Willett WC, Rimm EB, Stampfer MJ (1993). A prospective study of dietary calcium and other nutrients and the risk of symptomatic kidney stones. N Engl J Med.

[25] Hicks CW, Wang D, Matsushita K, Windham BG, Selvin E (2021). Peripheral neuropathy and all-cause and cardiovascular mortality in U.S. adults: a prospective cohort study. Ann Intern Med.

[26] Vasquez E, Batsis JA, Germain CM, Shaw BA (2014). Impact of obesity and physical activity on functional outcomes in the elderly: data from NHANES 2005–2010. J Aging Health.

[27] Hung JA, Li CH, Geng JH, Wu DW, Chen SC (2022). Dyslipidemia increases the risk of incident kidney stone disease in a large Taiwanese population follow-up study. Nutrients.

[28] Tan Z, Hong J, Sun A, Ding M, Shen J (2023). Causal effects of circulating lipids and lipid-lowering drugs on the risk of urinary stones: a mendelian randomization study. Front Endocrinol (Lausanne).

[29] Bobulescu IA, Lotan Y, Zhang J, Rosenthal TR, Rogers JT, Adams-Huet B, Sakhaee K, Moe OW (2014). Triglycerides in the human kidney cortex: relationship with body size. PLoS One.

[30] Cai C, Mai Z, Deng T, Zhao Z, Zhu W, Wen Y, Duan X, Wu W, Zeng G (2018). Impact of dyslipidemia on 24-h urine composition in adults without urolithiasis. Lipids Health Dis.

[31] Lee SC, Kim YJ, Kim TH, Yun SJ, Lee NK, Kim WJ (2008). Impact of obesity in patients with urolithiasis and its prognostic usefulness in stone recurrence. J Urol.

[32] Xu Z, Yao X, Duan C, Liu H, Xu H (2023). Metabolic changes in kidney stone disease. Front Immunol.

[33] Wilson PW (1990). High-density lipoprotein, low-density lipoprotein and coronary artery disease. Am J Cardiol.

[34] Morvaridzadeh M, Zoubdane N, Heshmati J, Alami M, Berrougui H, Khalil A (2024). High-density lipoprotein metabolism and function in cardiovascular diseases: what about aging and diet effects?. Nutrients.

[35] Tosheska Trajkovska K, Topuzovska S (2017). High-density lipoprotein metabolism and reverse cholesterol transport: strategies for raising HDL cholesterol. Anatol J Cardiol.

[36] Abdullah SM, Defina LF, Leonard D, Barlow CE, Radford NB, Willis BL, Rohatgi A, McGuire DK, de Lemos JA, Grundy SM (2018). Long-term association of low-density lipoprotein cholesterol with cardiovascular mortality in individuals at low 10-year risk of atherosclerotic cardiovascular disease. Circulation.

[37] Akin F, Altun I, Ayca B, Kose N, Altun I (2022). Associations of non-HDL-C and triglyceride/HDL-C ratio with coronary plaque burden and plaque characteristics in young adults. Bosn J Basic Med Sci.

[38] Siri-Tarino PW, Sun Q, Hu FB, Krauss RM (2010). Saturated fat, carbohydrate, and cardiovascular disease. Am J Clin Nutr.

[39] Liu AG, Ford NA, Hu FB, Zelman KM, Mozaffarian D, Kris-Etherton PM (2017). A healthy approach to dietary fats: understanding the science and taking action to reduce consumer confusion. Nutr J.

[40] Yiannakou I, Yuan M, Zhou X, Singer MR, Moore LL (2023). Dietary fat intakes, lipid profiles, adiposity, inflammation, and glucose in women and men in the Framingham offspring cohort. Front Physiol.

[41] Mietus-Snyder ML, Shigenaga MK, Suh JH, Shenvi SV, Lal A, McHugh T, Olson D, Lilienstein J, Krauss RM, Gildengoren G (2012). A nutrient-dense, high-fiber, fruit-based supplement bar increases HDL cholesterol, particularly large HDL, lowers homocysteine, and raises glutathione in a 2-wk trial. FASEB J.

[42] Wang Y, Xu D (2017). Effects of aerobic exercise on lipids and lipoproteins. Lipids Health Dis.

[43] Maxfield FR, Tabas I (2005). Role of cholesterol and lipid organization in disease. Nature.

[44] Bauer R, Brune B, Schmid T (2023). Cholesterol metabolism in the regulation of inflammatory responses. Front Pharmacol.

